# Case report: Severe nonketotic hyperglycinemia in a neonate without apparent seizures but concomitant cleft palate and cerebral sinovenous thrombosis

**DOI:** 10.3389/fped.2023.1155035

**Published:** 2023-08-08

**Authors:** Rapeepat Thewamit, Chaiyos Khongkhatithum, Lunliya Thampratankul, Wuttichart Kamolvisit, Arthaporn Khongkrapan, Duangrurdee Wattanasirichaigoon

**Affiliations:** ^1^ Division of Neurology, Department of Pediatrics, Faculty of Medicine Ramathibodi Hospital, Mahidol University, Bangkok, Thailand; ^2^ Division of Medical Genetics and Metabolism, Department of Pediatrics, King Chulalongkorn Memorial Hospital, Bangkok, Thailand; ^3^ Division of Medical Genetics, Department of Pediatrics, Faculty of Medicine Ramathibodi Hospital, Mahidol University, Bangkok, Thailand

**Keywords:** nonketotic hyperglycinemia, neonatal encephalopathy, cerebral venous sinus thrombosis, cleft palate, whole exome sequencing

## Abstract

Nonketotic hyperglycinemia (NKH) is in most cases a fatal inborn error of metabolism which usually presents during the neonatal period as encephalopathy and refractory seizures. The reported congenital anomalies associated with NKH included corpus callosal agenesis, club foot, cleft palate, and congenital heart disease. Here, we report a newborn who presented with encephalopathy without overt seizures, cerebral venous sinus thrombosis, and cleft palate. Electroencephalography showed a burst suppression pattern, which suggests the etiology could be due to a metabolic or genetic disorder. The amino acid analysis of plasma and cerebrospinal fluid showed elevated glycine. Whole exome sequencing identified a heterozygous c.492C > G; p.Tyr164Ter variant in exon 4 of the *GLDC* gene inherited from the patient's father. Further long-read whole genome sequencing revealed an exon 1–2 deletion in the *GLDC* gene inherited from the patient's mother. Additional analyses revealed no pathogenic variants of the cleft palate–related genes. The cleft palate may be an associated congenital anomaly in NKH. Regarding cerebral venous sinus thrombosis, we found a heterozygous variant (p.Arg189Trp) of the *PROC* gene, which is a common cause of thrombophilia among Thai newborns. A neonate with NKH could present with severe encephalopathy without seizures. A close follow up for clinical changes and further next generation sequencing are crucial for definite diagnosis in neonates with encephalopathy of unclear cause.

## Introduction

Nonketotic hyperglycinemia (NKH) (MIM 605899) is a rare inborn error of metabolism (1). The disorder is caused by mutations of both alleles in the genes encoding proteins in the glycine cleavage system (GCS), which consists of glycine decarboxylase (P-protein: *GLCD* gene), aminomethyltransferase (T-protein: *AMT* gene), dihydrolipoamide dehydrogenase (L-protein: *DLD* gene), and glycine cleavage system H-protein (H-protein: *GCSH* gene). Patients with severe NKH often present with lethargy, coma, hypotonia, apnea, hiccups, and refractory seizures before 2 weeks of age. Moreover, patients have a high mortality rate (85%) (2, 3). Patients with attenuated NKH usually present after 2 weeks of age and have variable neurodevelopmental outcomes, including impaired verbal communication with/without epilepsy (3, 4). There have been reports of congenital anomalies, including cleft palate (4.6% or 3/65 cases), club foot, corpus callosal agenesis, and cardiac abnormalities in patients with NKH (2, 5), albeit with an unclear pathogenic mechanism.

Herein, we describe a newborn with NKH who presented with encephalopathy without clinical seizures, cerebral venosinus thrombosis, and concomitant cleft palate. Additionally, extensive genetic analysis, including second and third generation sequencing plus cytogenomic microarray, were performed in an attempt to identify the genetic defect underlying NKH, cleft palate, and thrombosis.

## Case description

The patient was a 39-week gestational age male neonate, born via vaginal delivery following an unremarkable pregnancy with Apgar scores of 9 and 10 at 1 and 5 min, respectively, and a birth weight of 2,940 g. The patient was discharged to home on day of life (DOL) 2 with normal physical examination. At the age of 56 h (8 h after discharge), the patient was noted to become drowsy with a weak cry, necessitating an emergency visit and hospitalization at a local tertiary hospital. Physical examination revealed a temperature of 36.8°C, blood pressure of 75/30 mmHg, pulse rate of 140 beats/min, respiratory rate of 58 times/min, Glasgow Coma Score of 3 (E_1_V_1_M_1_), and pupils 2 mm, which were reactive to light. The muscle tone was hypotonic. Complete blood count, electrolytes, coagulogram (PT, PTT, fibrinogen level), arterial blood gases, and basic cerebrospinal fluid analysis were within normal limits. An amplitude-integrated electroencephalogram showed one suspicious electrographic seizure without a clinical seizure. Brain computer tomography (CT) showed minimal intraventricular hemorrhage at the bilateral occipital horn of lateral ventricles, thin subdural hemorrhage at the right tentorium, venous sinus thrombosis at the superior sagittal and transverse sinuses, and no congenital structural brain abnormalities. Phenobarbital was intravenously administered. The patient was intubated and transferred to our institution.

On DOL 3, at our hospital, the patient was comatose with apnea but without clinical seizure. Physical examination revealed cleft palate with no facial dysmorphic features, pupils 2 mm and normally reacting to light, the presence of doll eyes, generalized hypotonia, minimal spontaneous movement, deep tendon reflexes 1+, and absent Babinski signs. The Moro, suckling, and grasping reflexes were absent. The parents denied consanguineous marriage; however, they were from the same district. Both parents had no significant medical history of thrombosis. In addition, their first child had symptoms similar to the present patient and died on DOL 7 due to presumed infection.

**Figure 1:**
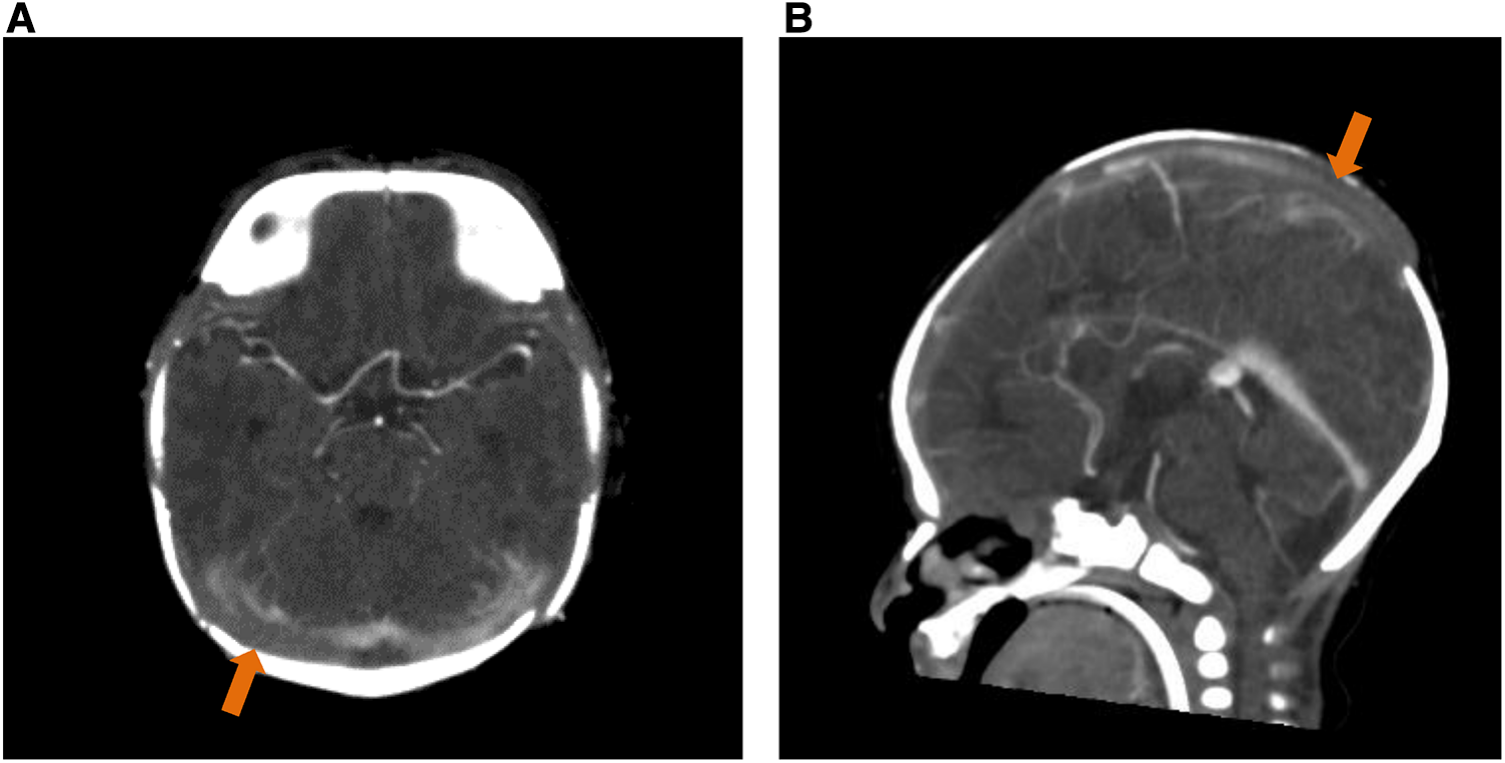
Contrasted brain CT of the patient. (**A**) Transverse sinus thrombosis. (**B**) Superior sagittal sinus thrombosis.

**Figure 2:**
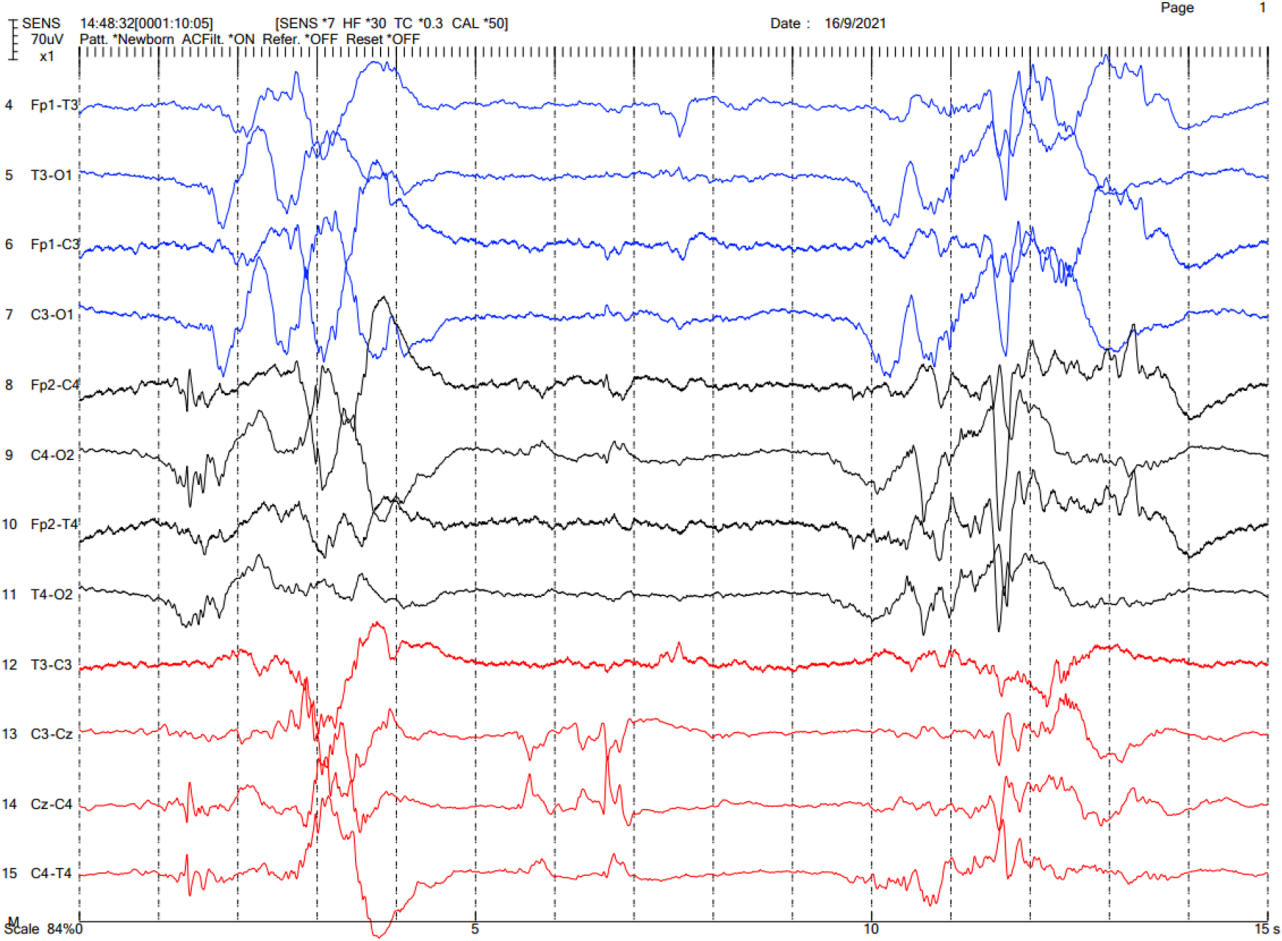
EEG of the patient showing the burst-suppression pattern.

The brain CT was reevaluated by our pediatric neuroradiologists, which showed cerebral venosinus thrombosis at the superior sagittal and transverse sinuses without evidence of cerebral edema or infarction (Figure 1). A brain MRI was not done. Protein C and protein S levels were not analyzed. Continuous electroencephalography (EEG) was performed, which showed a burst-suppression pattern without electrographic seizures (Figure 2). Pyridoxine 100 mg was intravenously administered and phenobarbital was continued; however, there was neither clinical nor EEG improvement. Given that the findings of brain CT could not explain the clinical findings of coma, we suspected that the etiology of coma and the burst suppression pattern on EEG could be an inborn error of metabolism or another rare genetic disease. Metabolic investigations were performed, which revealed a normal plasma ammonia level of 30 µmol/L (normal range 11–32 µmol/L), a mildly elevated lactate level of 2.5 µmol/L (normal range 0.5–2.2 µmol/L), and unremarkable urine organic acid profiles. Comprehensive metabolic screening (dry blood spot using tandem mass spectrometry) including acylcarnitine and amino acid profile analysis showed marked elevation of blood glycine (1,380 µmol/L; normal range 252–730 µmol/L). The CSF glycine level was 359 µmol/L (normal range 3.2–16.3 µmol/L). The patient's CSF/plasma glycine ratio was elevated at 0.31 (359/1,153 µmol/L). These results were consistent with severe NKH. Owing to the presence of cleft palate, cytogenomic microarray (CytoSNP-850 K, Illumina) was performed, which yielded no abnormality. Due to the severely poor prognosis of severe NKH, the parents were extensively counselled, and they decided to have the best supportive and symptomatic care for the patient. The family requested to have a peaceful terminal stage at their own place of residence. The patient passed away on DOL 7.

**Figure 3:**
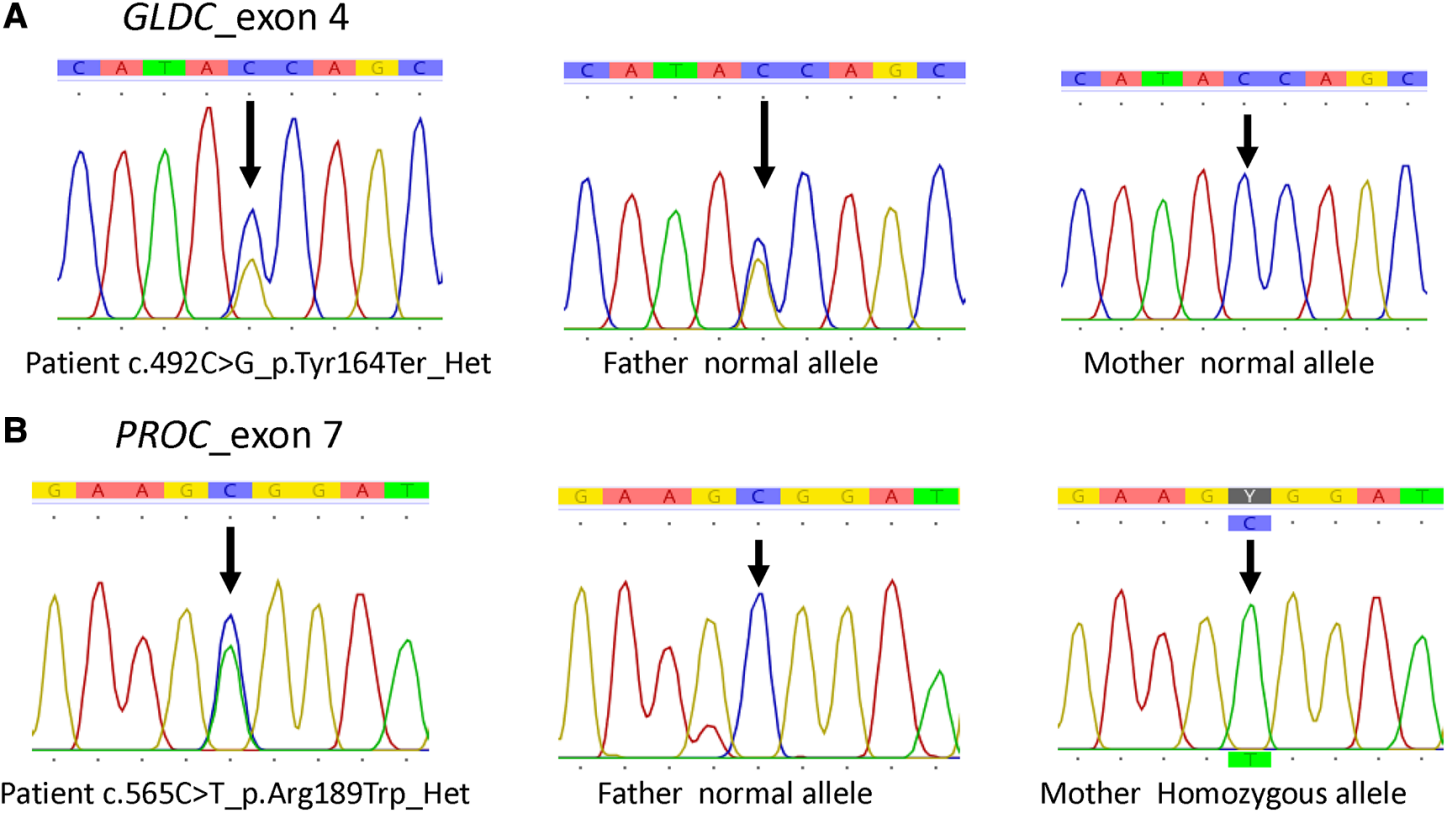
*GLDC* and *PROC* variants confirmed by Sanger sequencing of the family. (**A**) *GLDC* variants. (**B**) *PROC* variants.

Trio short-read whole exome sequencing (WES) with targeted gene analysis for hyperglycinemia (HP0002154: 19 genes) was performed, which revealed a heterozygous c.492C > G; p.Tyr164Ter (NM_000170.3) variant in exon 4 of the *GLDC* gene of the patient and the father, and no likely pathogenic/pathogenic variant in the *GLDC* gene of the mother (Figure 3A). Subsequently, long-read whole genome sequencing (WGS) was performed, which revealed exon 1–2 deletion in the *GLDC* gene of the patient and his mother. The methods for WES and WGS are provided in the Supplementary Material (6).

Additionally, the human phenotype ontology (HPO) terms cleft palate (HP0000175: 548 genes) and venous thrombosis (HP0004936: 73 genes; plus *PAI-1* gene) were analyzed to determine candidate variants underlying the cleft palate and cerebral venous thrombosis in the present patient. The analyses revealed no pathogenic/likely pathogenic variants of the cleft palate–related genes but a heterozygous pathogenic variant c.565C > T (p.Arg189Trp or Arg147Trp) in the protein C (*PROC*) gene (NM_000312.3) (Figure 3B). This *PROC* variant was inherited from his homozygous asymptomatic mother.

## Discussion

We confirmed a neonate with glycine encephalopathy and cleft palate due to compound heterozygous pathogenic variants (a nonsense mutation in exon 4 and a deletion of exons 1–2) in the *GLDC* gene. The coincidental finding of venous thrombosis in the patient was likely associated with the *PROC* Arg147Trp variant identified.

According to a clinical survey of NKH, seizures, apnea, and hiccups were found in 85%, 79%, and 95% of patients with severe neonatal NKH, respectively, whereas the only consistent finding was hypotonia (100%) (7). The seizures in these cases usually began within the first few hours to days of life (2, 3). Glycine can be both an excitatory and inhibitory neurotransmitter. Glycine can stimulate N-methyl-D-aspartate (NMDA) receptors in the hippocampus, cerebral cortex, and cerebellum, causing seizures. Glycine also binds to glycine receptors in the brainstem and spinal cord, leading to apnea, hypotonia, and hiccups. Our patient presented with encephalopathy without overt seizures, but with venous sinus thrombosis and cleft palate, complicating the differential diagnosis. However, subsequent findings of a burst suppression pattern on EEG helped narrow down the diagnosis. The definite diagnosis was obtained from metabolic and molecular genetic tests. As a matter of fact, the patient might have had overlooked clinical seizure because it is difficult to observe the seizures of neonates.

Once NKH is suspected, the confirmation can be quickly achieved by measuring CSF and plasma glycine levels and the CSF/plasma glycine ratio, as shown in this case. The deletion of exons 1–2 was not detected by short-read WES but was picked up by long-read WGS. Recent data has indicated that the combination of long-read WGS and conventional WES provide a better diagnostic yield and may be a more economic approach in daily practice in the near future, given the rapid decline in cost (8). WGS covers exons, introns, and the intergenic sequence; therefore, it expands the analysis to regions not detectable by WES. WGS can detect balanced and unbalanced translocation, complex structural variants, and large deletion/insertion. It has been shown that WGS increased the incremental diagnostic yield by up to 10% in patients with intellectual disabilities and by up to 25% in children with other undiagnosed disorders, as compared to WES (9, 10).

Cleft lip/palate has been reported in 4.6% (3/65) of patients with NKH; however, detailed clinical findings and etiopathogenesis of these cases were lacking (2). The presence of cleft palate in our patient led us to hypothesize that the cleft might represent (1) a coincidental syndromic or nonsyndromic cleft or a common cleft palate due to multifactorial disorder in which no abnormality of a single gene/chromosome could be detected; or (2) a true association between the orofacial cleft and NKH, due to an uncharacterized mechanism.

Given the normal result of the microarray and the absence of cleft-related genetic variants in the present patient, chromosomal abnormality and single gene disorder as the cause of the cleft anomaly was likely excluded, leaving the possibility of multifactorial disorder, which cannot be confirmed by genetic testing.

The mechanism of the possible “true” association between NKH and the orofacial cleft has been mysterious (2, 7); though a direct or indirect teratogenic effect of intrauterine hyperglycinemia is possible. We searched the protein-protein interaction database STRING (https://string-db.org/) and found that *SHMT2* (serine hydroxymethyltransferase), one among the top 10 proteins interacting with *GLDC* (Supplementary Material), was also listed as a cleft lip/palate–related gene, despite the absence of publication of *SHMT2* mutations as a cause of orofacial cleft. SHMT2 plays a role in glycine and tetrahydrofolate metabolism in which the tetrahydrofolate pathway is known to be linked to isolated orofacial cleft (11). Whether or not *SHMT2* is involved in hyperglycinemia-induced cleft palate requires further investigation.

Regarding cerebral venous sinus thrombosis, the risk factors associated with thromboembolism in neonates are central venous catheter, infection, asphyxia, polycythemia, and other predisposing factors. The heterozygous p.Arg189Trp variant of the *PROC* gene was likely the risk factor in the present patient. Protein C deficiency is a common risk factor of venous thromboembolism among Asian populations, including Thai, Chinese, and Japanese people (12–15). The disorder exhibits an autosomal dominant pattern with incomplete penetrance and variable expressivity (14). Homozygous individuals are more susceptible to develop venous thrombosis. The frequency of the Arg147Trp mutation in the *PROC* gene was prevalent in the Thai population; 9.5% for heterozygous and 2.7% for homozygous states (16). The Arg147Trp mutation increased the risk of cerebral venosinus thrombosis, with an odds ratio of 4.5 (95% CI: 1.6–12.8) for heterozygous and 43.3 (95% CI: 3.8–490.6) for homozygous states (16). A previous study found that both protein C and the functional level were decreased in Arg147Trp heterozygous individuals (14). An expression study of the *PROC* Arg147Trp variant in mammalian cell lines (HEK923) showed that the variant exhibited normal anticoagulant activity to Fva but a ∼3 times lower affinity for binding to endothelial protein C receptor (EPCR); thus the activation of this protein C variant on capillary endothelial cells could be potentially impaired and could contribute to the risk of developing venous thrombosis (14). Mutation of plasminogen activator inhibitor-1 (*PAI-1*), another thrombosis-related gene, was previously described in a neonate with NKH and cerebral venosinus thrombosis (17). The *PAI-1* variant was not found in our patient. We, therefore, proposed that poor feeding, dehydration, and the presence of the *PROC*: p.Arg147Trp variant together contributed to the cerebral thrombosis observed in the present neonate.

## Conclusion

We present a neonate with severe NKH in whom the presence of cerebral thrombosis together with cleft palate and initial absence of overt seizures complicated the diagnosis of NKH. A follow-up EEG looking for a burst suppression pattern and close monitoring for clinical changes are essential in neonates with encephalopathy of unclear cause, especially in countries lacking expanded newborn screening for inborn metabolic disorders.

## Data Availability

The datasets presented in this article are not readily available because of ethical and privacy restrictions. Requests to access the datasets should be directed to the corresponding author.
