# Supplementary material for: Case report: Severe nonketotic hyperglycinemia in a neonate without apparent seizures but concomitant cleft palate and cerebral sinovenous thrombosis
Source: Front Pediatr. 2023 Aug 8;11:1155035. doi: 10.3389/fped.2023.1155035 (PMC10442541; doi:10.3389/fped.2023.1155035)
Supplement: Supplementary file 1 [file Datasheet1.docx]

Supplementary Material

**Methods of short-read whole exome sequencing:**

Peripheral blood samples were obtained from the patient and the parents, for genetic testing, after written informed consent was given. DNA was extracted from the blood samples using a Gentra® Puregene® kit (QIAGEN®, Hilden, Germany).

Short-read whole exome sequencing of the individual’s specimen was performed on Illumina NovaSeq by Macrogen® **(**Seoul, Republic of Korea**)** and analyzed using previously described methods (1).

**Methods of long-read whole genome sequencing.**

***Sequencing***

Whole genome SMRTbell libraries were prepared by using SMRTbell Express Template Prep Kit 2.0 (P/N 100-938-900) and SMRTbell Enzyme Clean up Kit 2.0 (P/N 101-932-600) then eliminated fragment size below 8 Kb on BluePippin. Finally, SMRTbell libraries were sequenced on one smart cell of the Sequel II system after that raw subreads through the CCS workflow (PacBio SMRTLink version 10.0) to generate HiFi reads with a minimum estimated quality value (QV) of 20 (Phred scaled, corresponding to an accuracy of 99%).

***SV calling***

With 27.0 Gb of HiFi data, we performed alignment versus hg19 and SV calling on SMRT link program.

***Variant analysis***

Regarding the previous analysis, we found a missense allele on *GLDC* gene, so we specify to explore SV which is located on *GLDC* gene.

**Hyperglycinemia (HP:0002154): 19 genes**

| ALDH4A1 | COX8A | GLDC | GLYCTK | MMAA | MMUT | PCCA | PET117 | SLC30A10 | SUCLG1 |
| --- | --- | --- | --- | --- | --- | --- | --- | --- | --- |
| AMT | GCSH | GLRX5 | IRF6 | MMAB | NFS1 | PCCB | PSAT1 | SLC7A7 |  |

**Cleft palate (HP 0000175): 548 genes**

| ACBD5 | CD96 | DHCR24 | FGF8 | HYAL1 | MAP2K2 | PGAP1 | RAPSN | SIX5 | TCTN2 |
| --- | --- | --- | --- | --- | --- | --- | --- | --- | --- |
| ACTB | CDC45 | DHCR7 | FGF9 | HYLS1 | MAP3K7 | PGAP2 | RB1 | SLC10A7 | TCTN3 |
| ACTG2 | CDC6 | DHODH | FGFR1 | IFT140 | MAPK1 | PGAP3 | RBM10 | SLC18A3 | TELO2 |
| ADA2 | CDH1 | DLG3 | FGFR2 | IGBP1 | MAPRE2 | PGM1 | RBM8A | SLC25A19 | TFAP2A |
| AHI1 | CDH11 | DLL1 | FGFR3 | IGF2 | MASP1 | PHF8 | RECQL4 | SLC25A22 | TFE3 |
| ALG3 | CDKL5 | DLL3 | FGFRL1 | IL17RD | MBTPS2 | PHGDH | RET | SLC26A2 | TGDS |
| ALG9 | CDKN1C | DLX4 | FKBP14 | INPP5E | MECOM | PIEZO2 | RFWD3 | SLC2A10 | TGFB2 |
| ALX1 | CDON | DLX5 | FKRP | INPPL1 | MED12 | PIGG | RIC1 | SLC35D1 | TGFB3 |
| ALX3 | CDT1 | DMXL2 | FKTN | INTS1 | MED25 | PIGL | RIPK4 | SLC39A13 | TGFBR1 |
| ALX4 | CENPF | DOK7 | FLNA | INTU | MEGF10 | PIGN | RIPPLY2 | SLX4 | TGFBR2 |
| AMER1 | CEP120 | DONSON | FLNB | IPO8 | MEIS2 | PIGO | RNU4ATAC | SMAD3 | TGIF1 |
| AMMECR1 | CEP290 | DPH1 | FLRT3 | IQSEC2 | MEOX1 | PIGP | RPGRIP1 | SMAD4 | TMCO1 |
| ANKRD11 | CEP41 | DSE | FOXC2 | IRF6 | MESP2 | PIGQ | RPGRIP1L | SMARCA2 | TMEM107 |
| ANKRD17 | CEP57 | DSP | FOXE1 | ITGA8 | MID1 | PIGV | RPL11 | SMARCD1 | TMEM216 |
| ANOS1 | CHD7 | DUSP6 | FOXF1 | JUP | MIR140 | PIGW | RPL15 | SMC1A | TMEM231 |
| AR | CHN1 | DVL1 | FOXP2 | KANSL1 | MKKS | PIGY | RPL18 | SMC3 | TMEM237 |
| ARCN1 | CHRNA1 | DVL3 | FRAS1 | KAT5 | MKS1 | PITX1 | RPL26 | SMCHD1 | TMEM67 |
| ARHGAP31 | CHRND | DYNC2H1 | FTO | KAT6A | MSX1 | PLAG1 | RPL27 | SMO | TOPORS |
| ARID1B | CHRNG | DYRK1A | FZD2 | KAT6B | MSX2 | PLCB4 | RPL35 | SMOC1 | TP63 |
| ARID2 | CHST14 | EARS2 | G6PC3 | KATNIP | MUSK | PNKP | RPL35A | SMPD4 | TRIM8 |
| ARNT2 | CHST3 | EBP | GAD1 | KCNA1 | MYH3 | POGZ | RPL5 | SMS | TRIP13 |
| ARX | CHSY1 | ECEL1 | GATA1 | KCNH1 | MYL11 | POLA1 | RPS10 | SNRPB | TRPV4 |
| ASXL1 | CHUK | EDEM3 | GATA3 | KCNJ2 | MYMK | POLR1A | RPS15A | SNRPN | TRRAP |
| ATN1 | CILK1 | EDN1 | GDF11 | KCNK9 | MYOD1 | POLR1B | RPS17 | SON | TSR2 |
| ATP6V1B2 | COG1 | EDNRA | GDF3 | KCNN3 | NBN | POLR1C | RPS19 | SOX10 | TTC37 |
| ATR | COL11A1 | EFNB1 | GDF6 | KDM6A | NDNF | POLR1D | RPS23 | SOX2 | TTN |
| B3GALNT2 | COL11A2 | EFTUD2 | GJA1 | KIAA0586 | NEB | POLR2A | RPS24 | SOX3 | TUBB |
| B3GALT6 | COL2A1 | EIF2S3 | GJB2 | KIAA0753 | NECTIN1 | POLR3A | RPS26 | SOX6 | TWIST1 |
| B3GAT3 | COL4A1 | EIF4A3 | GLI2 | KIF14 | NEDD4L | POMGNT1 | RPS27 | SOX9 | TXNL4A |
| B3GLCT | COL9A1 | EIF5A | GLI3 | KIF7 | NEK1 | POMGNT2 | RPS28 | SPECC1L | UBE2T |
| B4GALT7 | COL9A2 | EPG5 | GMNN | KIFBP | NEUROD2 | POMK | RPS29 | SPEG | USP9X |
| B4GAT1 | COL9A3 | ERCC4 | GMPPB | KISS1 | NFASC | POMT1 | RPS7 | SPOP | VAX1 |
| B9D1 | COLEC10 | ERCC5 | GNAI3 | KISS1R | NFIX | POMT2 | RUNX2 | SPRY4 | WASHC5 |
| B9D2 | COLEC11 | ESCO2 | GNAO1 | KLHL41 | NIPBL | PORCN | RXYLT1 | STAC3 | WBP11 |
| BCOR | COMT | ESS2 | GNB1 | KMT2C | NODAL | POU1F1 | RYR1 | STAG2 | WDR11 |
| BCR | CPLANE1 | EXOC6B | GNB2 | KMT2D | NONO | PPP1CB | SALL4 | STAMBP | WDR26 |
| BGN | CPLX1 | EYA1 | GNRH1 | KRAS | NOTCH2 | PPP2R5D | SATB2 | STAT3 | WDR35 |
| BICRA | CREBBP | FAM149B1 | GNRHR | LARGE1 | NPHP1 | PPP3CA | SCARF2 | STIL | WNT3 |
| BIN1 | CRPPA | FAM20C | GP1BB | LARS2 | NSD2 | PQBP1 | SCN1B | SUFU | WNT4 |
| BMP4 | CSPP1 | FAM50A | GPC3 | LBR | NSMF | PRDM5 | SCN2A | SUMO1 | WNT5A |
| BMPER | CTBP1 | FANCA | GPC4 | LEMD3 | NSUN2 | PRKAR1B | SCUBE3 | SUPT16H | WNT7A |
| BPNT2 | CTCF | FANCB | GREB1L | LETM1 | NUAK2 | PROK2 | SEC23A | TAC3 | XRCC2 |
| BRAF | CTNND1 | FANCC | GRHL3 | LFNG | NUP107 | PROKR2 | SELENOI | TACR3 | XYLT1 |
| BRCA1 | CUL3 | FANCD2 | GRM7 | LHX4 | NUP88 | PROP1 | SEMA3A | TAPT1 | YAP1 |
| BRCA2 | CYP26C1 | FANCE | HAAO | LIG4 | OFD1 | PRR12 | SEMA3E | TBC1D24 | ZC4H2 |
| BRIP1 | CYTB | FANCF | HDAC8 | LMBRD1 | ORC1 | PRRX1 | SEPTIN9 | TBCE | ZEB2 |
| BUB1 | DAG1 | FANCG | HES7 | LMNA | ORC4 | PSAT1 | SETD5 | TBR1 | ZIC2 |
| BUB1B | DCC | FANCI | HESX1 | LMX1B | ORC6 | PTCH1 | SF3B2 | TBX1 | ZMPSTE24 |
| C2CD3 | DDX3X | FANCL | HMGA2 | LOXL3 | OTX2 | PTCH2 | SF3B4 | TBX15 | ZNF469 |
| CARS1 | DDX59 | FANCM | HNRNPK | LRRC32 | PALB2 | PTDSS1 | SHH | TBX2 | ZPR1 |
| CASK | DEAF1 | FEZF1 | HOXA2 | MAD2L2 | PAX3 | PTPN11 | SHMT2 | TBX22 | ZSWIM6 |
| CC2D2A | DEF6 | FGD1 | HOXD13 | MAF | PDE6D | RAD21 | SIAH1 | TBX4 |  |
| CCDC141 | DGCR2 | FGF10 | HS2ST1 | MAFB | PEX2 | RAD51 | SIK1 | TBX6 |  |
| CCDC22 | DGCR6 | FGF17 | HS6ST1 | MAMLD1 | PEX5 | RAD51C | SIX1 | TCOF1 |  |
| CCDC32 | DGCR8 | FGF20 | HSPG2 | MAP2K1 | PEX7 | RAI1 | SIX3 | TCTN1 |  |

**Venous thrombosis (HP 0004936): 73 genes and PAI-1 gene**

| ACVRL1 | CFH | EPOR | GDF2 | IRF2BP2 | MPI | NUMA1 | PROC | SCN5A | TET2 |
| --- | --- | --- | --- | --- | --- | --- | --- | --- | --- |
| ADA2 | CFI | F12 | GNAQ | JAK2 | MPL | P4HA2 | PROS1 | SERPINC1 | TGFB2 |
| AKT1 | CFTR | F13A1 | HABP2 | KCNK3 | MTHFR | PDE4D | PRSS1 | SERPIND1 | THBD |
| ALG6 | CITED2 | F2 | HBB | KCNN4 | MTRR | PDGFRA | PRSS2 | SH2B3 | THPO |
| BCOR | COX1 | F5 | HLA-B | KCNQ1 | MYH7 | PGM1 | PTEN | SLC2A10 | TLL1 |
| BMPR2 | COX3 | F8 | HLA-DRB1 | KIF11 | MYH9 | PIEZO1 | PTH1R | SLC4A1 | TNNI3 |
| C4A | CTLA4 | F9 | HRG | KIF20A | MYPN | PIGA | PTPN22 | SMAD4 | TNNT2 |
| CALR | CTNNB1 | FAS | IDH1 | LIPA | NFS1 | PIGM | RARA | SPINK1 | TP53 |
| CASR | CTRC | FGA | IDH2 | LPIN1 | NLRC4 | PLAT | RASA1 | STAT3 | TTR |
| CBS | CYTB | FGB | IFNGR1 | MEFV | NOTCH1 | PMM2 | RFT1 | STAT4 | UBA1 |
| CD46 | ENG | FGG | IL10 | MET | NPM1 | PRDX1 | RHAG | STAT5B | VHL |
| CD55 | EPAS1 | FLNC | IL23R | MMACHC | NPPA | PRKAR1A | RYR1 | TBL1XR1 |  |


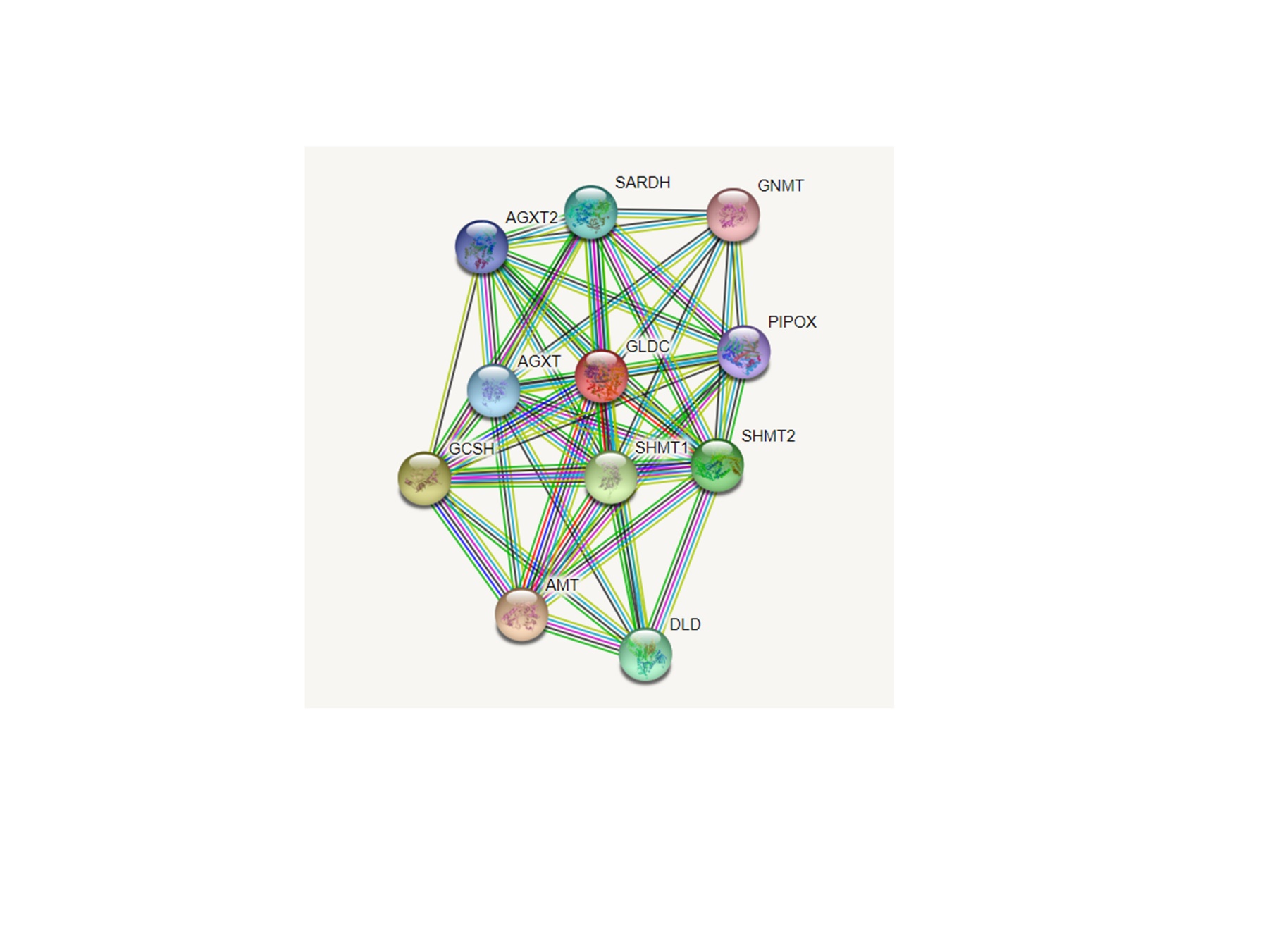


**Supplementary Figure 1.** Top 10 protein interacting with *GLDC* gene. Protein-protein interaction database, STRING (https://string-db.org/), indicating that *SHMT2* (serine hydroxymethyltransferase) which was also listed as a cleft lip/palate related gene, also interact with *GLDC* gene.

**Reference**

1. Thongpradit S, Jinawath N, Javed A, Jensen LT, Chunsuwan I, Rojnueangnit K, et al. Novel SOX10 Mutations in Waardenburg Syndrome: Functional Characterization and Genotype-Phenotype Analysis. Front Genet. 2020;11:589784.
